# Editorial: Image-based digital tools for diagnosis and surgical treatment: applications, challenges, and prospects 

**DOI:** 10.3389/fbioe.2025.1597238

**Published:** 2025-05-29

**Authors:** Laura Cercenelli, Adrian Elmi-Terander, Thomas Maal, Virginia Mamone, Francesca Manni

**Affiliations:** ^1^ eDIMES Lab - Laboratory of Bioengineering, Department of Medical and Surgical Sciences, University of Bologna, Bologna, Italy; ^2^ Department of Clinical Neuroscience, Karolinska Institutet (KI), Stockholm, Sweden; ^3^ 3D lab, Radboud University Nijmegen Medical Centre, Nijmegen, Netherlands; ^4^ Department of Computer Science, The EndoCAS Center for Computer‐Assisted Surgery, University of Pisa, Pisa, Italy; ^5^ Department of Electrical Engineering, Eindhoven University of Technology (TU/e), Eindhoven, Netherlands

**Keywords:** 3D modeling and printing, augmented (virtual) reality, radiomics, image-based diagnosis, surgical planning and simulation

## Introduction

The advent of image-based digital tools has revolutionized the landscape of modern medicine, particularly in diagnosis and surgical treatment. Advances in deep learning, computer vision, 3D modeling, 3D printing and Augmented Reality (AR) have significantly enhanced the precision and efficacy of medical procedures, reducing human error and improving patient outcomes.

The articles collected within this Research Topic explore various facets of these emerging technologies, highlighting their applications, challenges, and prospects.

The collected studies can be grouped into the following focus areas of the Research Topic.

## Innovations in medical image segmentation and radiomics

Deep learning-based segmentation has emerged as a cornerstone in medical image analysis. Also radiomics, i.e., the extraction of quantitative features from medical images, has gained traction as a tool for enhancing diagnostic accuracy.

The study by Abidin et al. presents a comprehensive review of brain tumor segmentation using multi-modal MRI and deep learning techniques. Their survey categorizes state-of-the-art models into CNN-based, transformer-based, and hybrid architectures, underscoring the strengths and weaknesses of each approach.

Mao et al. introduce a 2D medical image segmentation framework called Progressive Learning Network (PL-Net), which optimizes medical image segmentation by integrating coarse-to-fine semantic learning without increasing computational complexity.

Sun et al. present the innovative DA-TransUNet framework, which incorporates a Dual Attention Block (DA-Block) — combining position and channel attention—into a Transformer-enhanced U-Net architecture. This tailored design allows to improve segmentation accuracy particularly for high-detail medical images by refining feature extraction and filtering out irrelevant information.

The study by Lv et al. proposed an automated method for mandibular canal segmentation using a transformer-based neural network with cl-Dice loss and pixel-level feature fusion to improve accuracy. Their approach addresses challenges like sample imbalance and unclear boundaries through mandibular foramen localization, contrast enhancement, and pre-training with Deep Label Fusion on synthetic datasets. The method achieved state-of-the-art results, demonstrating high precision and robustness in 3D mandibular canal localization.

Jia et al. explore the application of radiomics in optimizing diagnosis and surgical planning for chronic osteomyelitis. Their findings indicate that an expanded region of interest (ROI) in MRI scans improves predictive performance, offering valuable insights for precision medicine approaches.

Finally, the study by Chen et al. reviews recent advances in retinal vessel quantification technology based on fundus imaging, highlighting its key role in detecting and monitoring ocular and systemic diseases. It focuses on how innovations in imaging and AI have enhanced diagnostic accuracy, offering clinicians and researchers an updated overview of its clinical applications.

Across these studies, a shared emphasis emerges on refining feature extraction and dealing with data complexity through attention mechanisms or radiomic modeling. Several approaches tackle the ongoing challenges of data imbalance and annotation scarcity, particularly in high-detail segmentation tasks. Additionally, transformer-based methods consistently appear as a unifying trend aimed at capturing long-range dependencies and improving contextual awareness.

## Advances in 3D imaging, modeling and printing for surgical planning and regenerative medicine

The rapid evolution of 3D imaging, modeling and printing technologies is creating unparalleled opportunities to optimize surgical planning, improve patient outcomes, and driving progress in regenerative medicine.

In this context, Henckel et al. advanced the validation of low-dose 3D-CT imaging for acetabular implant orientation in orthopedic surgery. Their findings confirmed that 3D-CT is a highly accurate and precise modality for measuring cup inclination and version in total hip arthroplasty, outperforming conventional 2D CT methods. The results support 3D-CT as a standard for post-operative evaluation and surgical system optimization.

Similarly, Wu et al. developed novel 2D and 3D CT-based injury models for predicting the risk of femoral neck fracture in patients with femoral head fractures. Their quantitative parameters—such as percentage of maximum defect length and fracture area—showed high diagnostic accuracy and can serve as reliable predictors of fracture risk. This approach provides clinicians with valuable decision-making tools in trauma management and preoperative risk assessment.

In the domain of 3D printing, Capellini et al. presented a brief research report on the application of 3D printed models for the management of complex congenital heart disease. Their work underscores how physical models derived from high-resolution MRI and CT datasets allow for a detailed and tactile understanding of intricate pediatric cardiac anatomies. Among the techniques explored, selective laser sintering (SLS) proved to be the most cost-effective and time-efficient solution.

Pisani et al. explored bioartificial scaffolds produced using solvent casting, electrospinning, and 3D printing within the field of regenerative medicine. Their findings demonstrate that hybrid scaffolds—merging various fabrication methods—achieve excellent cell viability and mechanical properties similar to native soft tissues.

Moving to veterinary surgery, Chambers et al. evaluated a custom 3D-printed cutting guide for canine caudal maxillectomy, finding that it improved surgical accuracy for both experienced and novice surgeons. While it slightly increased procedure time, the enhanced precision, especially in achieving oncologic margins, suggests that such digital tools can assist less experienced surgeons and improve outcomes in veterinary oncology.

The studies in this area collectively highlight the growing reliance on patient-specific models, whether for orthopedic planning and post-operative evaluation, trauma prediction, or surgical simulation. A consistent theme is the shift toward integrating anatomical fidelity with fabrication feasibility—balancing precision and efficiency. Moreover, the translational relevance of these models is emphasized both in human and veterinary medicine, underscoring the broad applicability of 3D digital workflows.

## Augmented reality in surgery and patient education

The integration of AR into surgery and patient education is another transformative development improving surgical accuracy and enriching communication between clinicians and patients.

Nasir et al. provide a systematic review of AR applications in orthopedic and maxillofacial oncological surgeries, detailing its potential to enhance precision through improved visualization. The study emphasizes the need for further clinical validation and the integration of external navigation systems to improve accuracy.

Similarly, Urlings et al. investigate the role of AR in patient education for intracranial aneurysms, highlighting how immersive visualizations can enhance patient understanding and shared decision-making.

Both studies highlight the visualization benefits of AR, while also pointing to common implementation hurdles—particularly the challenges of integrating AR with surgical navigation systems and the need for robust clinical validation. Whether applied in the operating room or during patient consultations, AR consistently emerges as a powerful tool for enhancing spatial understanding. However, technical limitations continue to hinder its broader adoption in clinical settings.

## Conclusion and future directions

The contributions within this Research Topic underscore the transformative impact of digital imaging tools in diagnosis and surgical treatment. From AI-driven segmentation techniques to AR-enhanced surgical interventions, these advancements promise a future where precision medicine is more accessible, efficient, and patient-centered.

Across all sections, a shared trajectory emerges: the shift toward more personalized, data-driven, and visually guided approaches to healthcare. While each study presents specific technological innovations, they collectively reflect a broader trend of converging digital methodologies—such as the use of transformers in segmentation, patient-specific 3D models for planning, and immersive AR for both surgeons and patients. These tools not only enhance accuracy and confidence in clinical decision-making but also foster interdisciplinary collaboration among clinicians, engineers, and data scientists.

Despite these promising advancements, challenges remain on the path to real-world clinical translation. In AI-based segmentation tools, the major issues deal with computational efficiency, the need for large, annotated datasets, and model interpretability. In surgery, AR faces limitations in real-time tracking accuracy and workflow integration. For 3D modeling and printing, the translation of digital data into physical models requires standardized protocols and cost-effectiveness evaluations for broader clinical adoption.

Moreover, regulatory hurdles and data governance policies pose significant obstacles, especially for AI applications and patient-specific devices. Bridging the gap between innovation and implementation will require not only technical refinement but also rigorous validation, regulatory clarity, and integration into clinical training and reimbursement frameworks.

The insights from the included studies suggest that an integrated ecosystem—where data, tools, and expertise converge—can accelerate the transition from research innovation to bedside impact. Continued research and cross-disciplinary collaborations between computer scientists, engineers, radiologists, and surgeons will be essential for translating innovations from the lab into clinical practice and for achieving the full potential of image-based digital tools in delivering safer, more effective, and tailored patient care.

